# Heavy metal pollution and ecological risk assessment in brownfield soil from Xi’an, China: An integrated analysis of man-land interrelations

**DOI:** 10.1371/journal.pone.0241398

**Published:** 2020-11-02

**Authors:** Siqi Liu, Luyao Wang, Chao Guo

**Affiliations:** 1 Shaanxi Provincial Land Engineering Construction Group Co., Ltd., Xi’an, China; 2 Institute of Land Engineering and Technology, Shaanxi Provincial Land Engineering Construction Group Co., Ltd., Xi′an, China; 3 Key Laboratory of Degraded and Unused Land Consolidation Engineering, the Ministry of Natural Resources, Xi′an, China; 4 Shaanxi Provincial Land Consolidation Engineering Technology Research Center, Xi′an, China; Institute for Advanced Sustainability Studies, GERMANY

## Abstract

The core of urbanization is land use change, resulting in the urban sprawl and urban population explosion. The problem of land resources shortage and ecological environment destruction has become increasingly prominent. Land use change and human activities can directly lead to urban soil pollution. This study analyzed the concentration of Cd, Cr, Cu, Ni, Pb, Zn, Hg and As in the original site of Xi’an chlor-alkali chemical plant, which was know as a brownfield. The results showed the concentrations of Hg, Pb and Zn in research areas were obviously higher than soil background value. Through pollution index (PI) method and Geo-accumulation Index (I_geo_) method, totally 26 sample points in different areas (A, B, C, D) were classified into different pollution degrees. The CPI results indicated 9 sample points were heavily polluted, accounting for 34.6% of the total. Among them, 6 out of 9 were located in area A. 12 samples points were not polluted. The average I_geo_ values of single heavy metal were arranged in the order of Hg (1.83) > Zn (1.26) > Pb (0.33). The pollution of Hg was relatively serious and extensive, especially in area A. It was mainly because of the historical pollution produced by chemical plant. The pollution of Pb in each point was quite different. Mainly influenced by automobile related activities, I_geo_(Pb) in sample point 15 and 16 were all beyond 4.00. The average potential ecological risk (PER) of each area was in the order of A (1428) > B (297) > D (249) > C (163). The ecological risk was mainly determined by previous industrial production and present human activity at the same time. People and land are interdependent and interactive. The understanding on the mechanism of man-land interralations, regarding to urban land use and ecological environment, will promote urban sustainability.

## 1. Introduction

Urbanization and industrialization are crucial to realize modernization. This process strongly changes the economic structure and ecological system structure of urban areas, and causes a series of ecological environment problems [1–3]. The essence of these problems is the deterioration of man-land interrelations. Human and ecological environment are interdependent and mutually restricted. Harmonious man-land interrelations will greatly contribute to urban sustainable development [4–6]. The urbanization in China is remarkable as the drastic land changes and significant conflicts of development among economy, social security and environment [7,8]. Since 2013, China formally established a new urbanization development strategy of "Human-Oriented". It indicated that the development of urbanization in China had been gradually changed from quantity expansion type to quality promotion type, mainly reflected in the optimization of ecological environment, urban functional layout and industrial structure, as well as the improvement of social security system. Intensification and sustainability of land use became a trend and were widely accepted [9,10].

Urban soil is an important part of urban ecological system, which is the natural foundation of urbanization construction. It is closely related to urban development and public health [11,12]. A large number of pollutants produced by rapid industrialization and modern transportation eventually enter urban soil directly or indirectly. As the final receptor of most pollutants, soil has a high potential pollution risk [13]. In recent years, various kinds of soil pollution events have occurred in China frequently, such as endemic arsenism. It indicates that urban soil is suffering from severe pollution and human health is seriously threatened as a result.

Heavy metal pollution is a very common soil pollution type, especially in urban area. The sources of heavy metal pollution are various, and the situation of multi heavy metal pollution is widespread and quite serious [14]. Industrial production, human activities and transportation are all the direct causes leading to heavy metal pollution in urban soil [15]. Some researchers suggested that Pb pollution in urban soil is due to the combustion of leaded gasoline through Pb isotope composition analysis [16]. Heavy metal pollution including Cu, Cd, Pb, Zn, Ni, As were mainly located surrounding historical high-pollution chemical plants through geographical information system (GIS) technology [17]. Heavy metal pollution is persistent, latent and irreversible with obvious agglomeration effect in a certain area, such as abandoned industrial site (brownfield). Brownfield includes abandoned, unused or underutilized industrial or commercial land, which is complicated by actual or potential pollution [18]. In China, 34.9% of the soil sample points in abandoned industrial site are contaminated. Among them, more than a third of the points are seriously contaminated [19].

Xi'an, as an ancient capital and an old industrial base in China, plays an important role in Belt and Road strategic plan. Since 1950s, Xi'an has gradually developed into an important industrial city in western China. Heavy industry enterprises that are easy to produce heavy metal pollution, such as iron and steel plants, metallurgical machinery plants, thermal power plants and petrochemical plants, are mainly located in the west of Xi’an city. In recent years, with the optimization of industrial structure and the adjustment of urban land layout, many industrial factories in Xi'an have been demolished or moved to the outskirts of the city [20]. The industrial sites have a great threat to residents’ health.

The pollution of brownfield is complex and dynamic, which is accompanied by urban development and land use change. Many researchers believe that the key of brownfield redevelopment is to maintain dynamic balance of man land relationship. It is necessary to fully understand the present pollution condition as well as to prevent these sites from secondary soil pollution in the future urban development. Green addressed the significance of active participation of different groups [21]. Martinat found that brownfield redevelopment was an opportunity to provide urban land and to create jobs for new industries through the field survey of 28 brownfield sites of Karvina, in the Czech Republic [22]. Furthermore, the identification of pollution source is the precondition of brownfield redevelopment, which is quite complicated. Yang analyzed the spatial distribution of heavy metals of Linfen in China, and found that Cu, As and Pb probably originated from the similar anthropogenic sources [23]. Similarly, Lv found that Hg was dominated by land use and attributed to anthropogenic emissions using GIS-based data [24]. Brownfield development should not only ensure human and ecological health, but also meet the needs of urban development. This research focused on the dynamic changes of brownfield soil pollution. Through on-site investigation, heavy metal pollution and ecological risk assessment, this study aimed at (1) analyzing the concentration of Cd, Cr, Cu, Ni, Pb, Zn, Hg, As in 26 sample points of research areas; (2) using pollution index (PI) method and Geo-accumulation Index (I_geo_) method to evaluate the pollution degree; (3) using Potential Ecological Risk (PER) assessment model to evaluate ecological risk in different areas; (4) specifying the sources of pollution based on the land use pattern and human activities; (5) giving some advices based on man-land interrelations and urban sustainability.

## 2. Materials and methods

### 2.1. Study area and soil sampling

The original site of Xi’an chlor-alkali chemical plant is located at No. 356, Kunming Road, Yanta District, Xi'an City, which was founded in 1958. It was in the west of Xi’an at latitude 34°14'54"—34°15'8", longitude 108°51'20"—108°51'46". It was a large-scale chlor-alkali chemical enterprise mainly engaged in the production of caustic soda, PVC, calcium carbide, and a variety of other chemical raw materials. In 2009, it was moved to Jingwei Industrial Park of Xi'an Economic and Technological Development Zone as a whole, and the original plant site of Xi'an Chemical Plant was shut down in 2012. With the expansion of the city, the land around the site had been gradually changed from rural areas to urban functional areas, such as residential areas. After the industrial factory was demolished, the land was managed by the government and belonged to the urban public land, which was used for public parking, car repair and logistics storage. The site was open and unrestricted. It was allowed to carry out reasonable and legal activities within the site. Therefore, no permission was required in this area.

**Fig 1:**
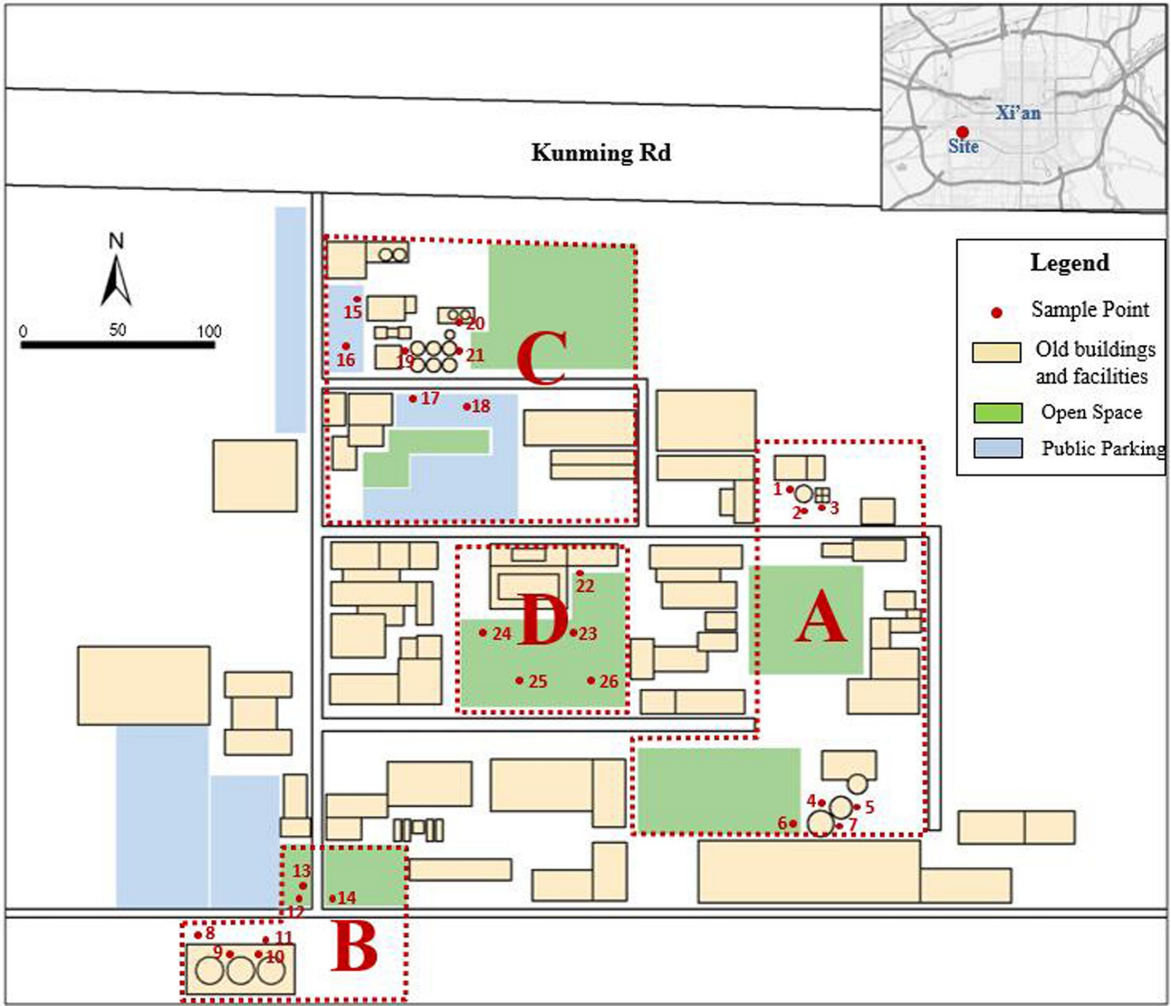
Study area and soil sample points.

The sampling points of this research were arranged by 3S technology, which is a combination of geographic information system (GIS), remote sensing (RS) and global positioning system (GPS). Through the collection of historical data and field survey, the suspected pollution areas in the plant were identified. The research area was divided into areas A, B, C and D based on different industrial zoning, present land use pattern and spatial characteristics. Totally 26 soil sample points were selected (Fig 1). According to Technical Guidelines for Environmental Site Monitoring (HJ25.2–2014) in China, each sample point was divided into nine equal grids and nine subsamples were obtained in the center of each grid. These nine subsamples were mixed as a composite soil sample and then divided into three parallel samples. As a result, there were totally 78 surface composite soil samples in this research. During sampling, the sundries on the surface of soil were removed, and the sampling depth was 0–20 cm.

### 2.2. Analytic methods

This research adopted both on-site portable quick detection and laboratory chemical analysis to analyze the concentration of heavy metals in soil, including Cd, Cr, Cu, Ni, Pb, Zn, Hg, As. These eight heavy metals are widely distributed and have different degrees of harm to human health [25]. This process mainly includes two stages. First, based on X-ray fluorescence (XRF)spectrometry, portable testing equipment (SciAps, X-200) was utilized to carry out extensive test of soil on site. The test meets the requirements of EPA method 6200. The detectable elements are shown in Table 1. This stage can identify areas with potential pollution risks, which can realize qualitative and semi-quantitative analysis of samples. Second, soil samples were collected in key areas with potential pollution risks according to the result of first stage. After complete chemical digestion (US EPA method 3050B), ICP-MS (Agilent, 7700) and Atomic Fluorescence Spectrometer (JINSUOKUN, SK-2003AZ) were utilized to analyze the concentration of Cd, Cr, Cu, Ni, Pb, Zn, Hg, As. National standard materials for soil composition analysis (GSS-8) were used for analytic quality control (QC).

**Table 1:** Standard element package in Geo-Env Soil Mode of SciAps X-200.

Mode	Detectable Elements (Beam 1)	Detectable Elements (Beam 2)	Detectable Elements (Beam 3)
Soil	Ti, V, Cr, Mn, Fe, Co, Ni, Cu, Zn, As, Se, Sr, Rb, Zr, Mo, W, Tl, Hg, Pb, Bi	Mg, Al, Si, P, S	Ag, Cd, Sn, Sb, Ba

### 2.3. Pollution Index (PI) method

Single pollution index (SPI) method is an important evaluation method of single heavy metal. By setting appropriate evaluation standard or reference value of heavy metal, we can clearly judge the ratio between sample and reference value, and evaluate heavy metal pollution in soil scientifically, which is widely used in environmental pollution investigation and evaluation research. The formula is:

$$P_{i}=C_{i}/S_{i}$$ (1)

C_i_ is the measured concentration of heavy metals in the soil, S_i_ is the evaluation standard or reference value of heavy metals in the soil, P_i_ is the SPI. The evaluation standard of heavy metals is in accordance with the secondary standard (pH: 6.5–7.5) in the Soil Environmental Quality Standard. Additionally, relevent criteria in Risk Control Standard for Soil Contamination of Development Land (GB36600-2018) complemented this study.

Comprehensive pollution index (CPI) method is one of the most commonly used methods for multi heavy metals pollution evaluation at present. This method compares and analyzes the maximum value and average value of multiple factors, and the results can directly reflect the severity of comprehensive pollution [26,27]. The formula is:

$$P_{c}=\sqrt{\frac{P_{imax}^{2}+P_{iave}^{2}}{2}}$$ (2)

P_imax_ is the maximum value of SPI; P_iave_ is the arithmetic mean value of SPI, and P_C_ is the CPI. According to the value of P_c_, the soil pollution degree is divided into five levels, as shown in Table 2.

**Table 2:** Pollution grades of CPI.

Levels	P_c_	Pollution Grade
I	P_c_ ≤ 0.7	Clean
II	0.7< P_c_ ≤1	Low
III	1< P_c_ ≤2	Moderate
IV	2< P_c_ ≤3	High
V	P_c_ > 3	Significantly High

### 2.4. Geo-accumulation Index (I_geo_) method

The Geo-accumulation Index (I_geo_) method is proposed based on the study of pollution levels in sediments, which can comprehensively take the influence of human activities and natural distribution characteristics into consideration [28]. The formula is:

$$I_{geo}={log}_{2}\left[\frac{C_{n}}{{K\times B}_{n}}\right]$$ (3)

C_n_ is the concentration of chemical element n in the sediment; B_n_ is the geochemical background value of the element in the sediment; K is the coefficient (generally k = 1.5) taken to consider the variation of the background value that may be caused by the difference of rocks in different places. The evaluation standard of I_geo_.is shown in Table 3.

**Table 3:** Pollution grades of I_geo_.

I_geo_	Levels	Pollution Grade
I_geo_ < 0	0	Not polluted
0 ≤ I_geo_ < 1	1	Not Polluted to moderately polluted
1 ≤ I_geo_ < 2	2	Moderately polluted
2 ≤ I_geo_ <3	3	Moderately polluted to heavily polluted
3 ≤ I_geo_ < 4	4	Heavily polluted
4 ≤ I_geo_ < 5	5	Heavily polluted to extremely polluted
5 ≤ I_geo_	6	Extremely polluted

### 2.5. Potential Ecological Risk(PER) assessment model (Hakanson)

The potential ecological risk (PER) assessment model was proposed by Hakanson, a Swedish scientist, which was used to evaluate heavy metal pollution [29]. This method fully considers the toxicity difference and ecological sensitivity of various heavy metals, introduces the concept of toxicity response parameters, and classifies quantitatively the potential pollution degree of heavy metals to the ecological environment. The calculation formula is as follows(4)-(6):

$$C_{f}^{i}=C_{s}^{i}/C_{n}^{i}$$ (4)

$$E_{r}^{i}=T_{r}^{i}\times C_{f}^{i}$$ (5)

$$RI=\sum_{i=1}^{n}E_{r}^{i}$$ (6)

C_f_^i^ is the pollution coefficient of heavy metals; C_s_^i^ is the measured concentration of heavy metals in soil; C_n_^i^ is the reference ratio needed for this study. In order to show the specificity of different regions, the reference ratio is determined by combining the soil background value of Xi'an city and the representative value of background value of soil elements in China (1990); E_r_^i^ is the PER index of single heavy metal; T_r_^i^ is the toxicity response parameter of single heavy metal. This model was based on the standardized heavy metal toxicity response coefficient developed by Hakanson, as shown in Table 4; RI is the potential ecological risk index of various heavy metals, and the comprehensive PER level classification is shown in Table 5.

**Table 4:** The reference ratio (C_n_^i^) and toxicity response parameter (T_r_^i^) of heavy metals in soil.

Element	Cd	Cr	Cu	Ni	Pb	Zn	Hg	As
C_n_^i^	0.2866	70.8	26.0	64.0	38.8	31.2	0.065	11.2
T_r_^i^	30	2	5	5	5	1	40	10

**Table 5:** Comprehensive potential ecological risk (PER) levels classification.

RI	Comprehensive PER level
RI<150	Low
150≤RI<300	Moderate
300≤RI<600	High
600≤RI	Significantly High

## 3. Results

There were 26 soil sample points in total. The mean values of three parallel samples were used to describe the concentration of heavy metals at each point. The concentration of Cd, Cr, Cu, Ni, Pb, Zn, Hg and As had been tested in each sample point. The results are shown in Table 6. The detection rate of Cd, Cr was relatively low, which was 15% and 27% respectively. Therefore these results were not included in the statistics. The mean concentrations of Cu, Ni, Pb, Zn, Hg and As were 46.27 mg·kg^-1^, 69.54 mg·kg^-1^, 205.98 mg·kg^-1^, 140.88 mg·kg^-1^, 0.81 mg·kg^-1^, 11.40 mg·kg^-1^ respectively. The values of Hg, Pb and Zn were significantly higher than the background values of Xi'an soil (the background values of Hg are 0.065 mg·kg^-1^, Pb are 38.8 mg·kg^-1^, Zn are 31.2 mg·kg^-1^), which was probably influenced by human activities. The coefficients of variation (CV) of Cu, Ni, Pb, Zn, Hg were more than 50%. The elements with the largest standard deviation (SD) and coefficient of variation (CV) was Pb, reaching 408.04 and 198.09% respectively, reflecting that the concentration of Pb in various points was quite different. The element with the smallest standard deviation (SD) was Hg, which was 0.95. The element with the lowest coefficient of variation (CV) was As, which showed the concentrations in different samples were similar numerically.

**Table 6:** Descriptive statistics of heavy metal concentrations at 26 sample points.

Heavy Metals	Cd	Cr	Cu	Ni	Pb	Zn	Hg	As
**Mean** (mg·kg^-1^)	/	/	46.27	69.54	205.98	140.88	0.81	11.40
**Median** (mg·kg^-1^)	/	/	30.27	42.57	42.94	103.34	0.35	11.55
**SD**	/	/	44.32	80.57	408.04	130.21	0.95	2.46
**Minimum** (mg·kg^-1^)	/	/	13.70	30.35	11.56	53.74	0.04	4.50
**Maximum** (mg·kg^-1^)	0.36	171.56	189.49	418.51	1788.10	689.55	2.95	16.70
**CV** (%)	/	/	95.80	115.86	198.09	92.43	118.31	21.54

*SD = Standard deviation; CV = Coefficient of variation.

*/ indicates the results are not included in statistics. The detection rate of Cd, Cr is 15% (4/26) and 27% (7/26) respecitively.

**Fig 2:**
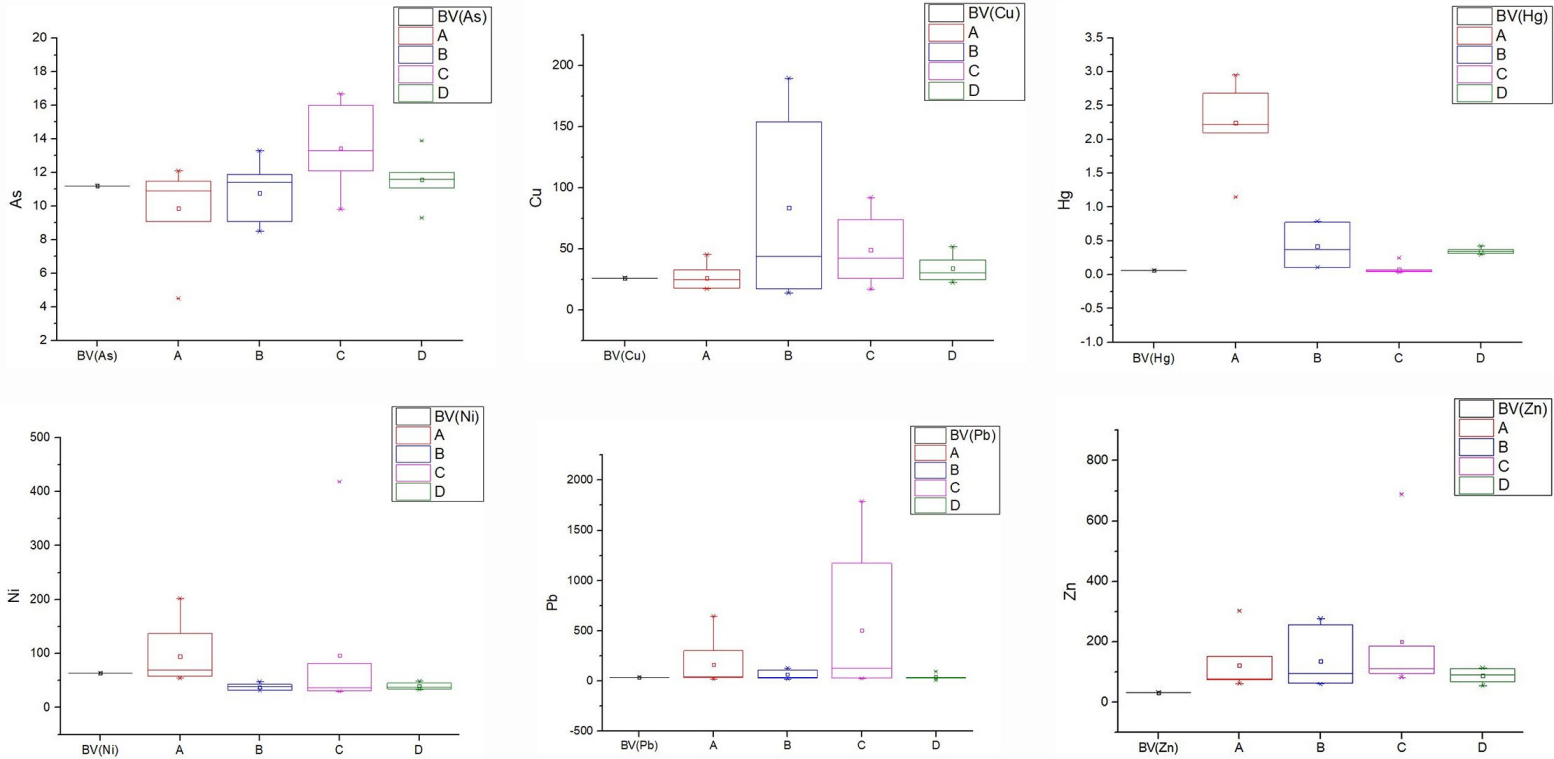
Box plots of heavy metals in Area A, B, C, D and background value. *BV = Background value, the BV of Cu, Ni, Pb, Zn refer to the soil background of Xi’an, Hg, As refer to the representative values of Background Values of Soil Elements in China (1990).

According to Fig 2, the types and degrees of heavy metal pollution in different study areas were quite different. The mean concentrations of Pb, Zn and Hg in all study areas (A, B, C, D) were higher than the background value. The concentration of Hg in Zone A, Cu in zone B and Pb in Zone C were significantly higher than the background value, in which the fluctuation range of Cu and Pb was larger, and the difference between the sample points was significant. The concentration of Hg stayed in a stable and high range of value, which reflected that this regularity was generally existed in the whole study areas. The concentration of heavy metals in area D was relatively small, and their fluctuation was not obvious, which was close to the soil condition of natural background.

The results of CPI are shown in Table 7. Nine samples were heavily polluted (grade V), accounting for 34.6% of the total; two samples were lightly polluted (grade III), accounting for 7.7% of the total; three samples were slightly polluted (grade II), accounting for 11.5% of the total; and 12 samples were non polluted (grade I), accounting for 46.2% of the total. It can be found that the heavily polluted area was mainly concentrated in area A, while the non polluted area was mainly concentrated in area D. Zone B showed slight pollution with different degrees. The pollution degree of different sample points in area C was quite different, among which the point pollution near public parking area was more serious.

**Table 7:** The results of CPI.

Sample Point	P_c_	Levels	Sample Point	P_c_	Levels	Sample Point	P_c_	Levels
1	1.44	III	10	0.69	I	19	0.12	I
2	5.04	V	11	0.70	II	20	0.16	I
3	5.12	V	12	0.18	I	21	0.35	I
4	9.22	V	13	1.06	III	22	0.24	I
5	8.20	V	14	0.30	I	23	0.17	I
6	5.22	V	15	4.10	V	24	0.19	I
7	6.14	V	16	9.40	V	25	0.31	I
8	0.81	II	17	0.78	II	26	0.25	I
9	0.19	I	18	18.55	V			

**Fig 3:**
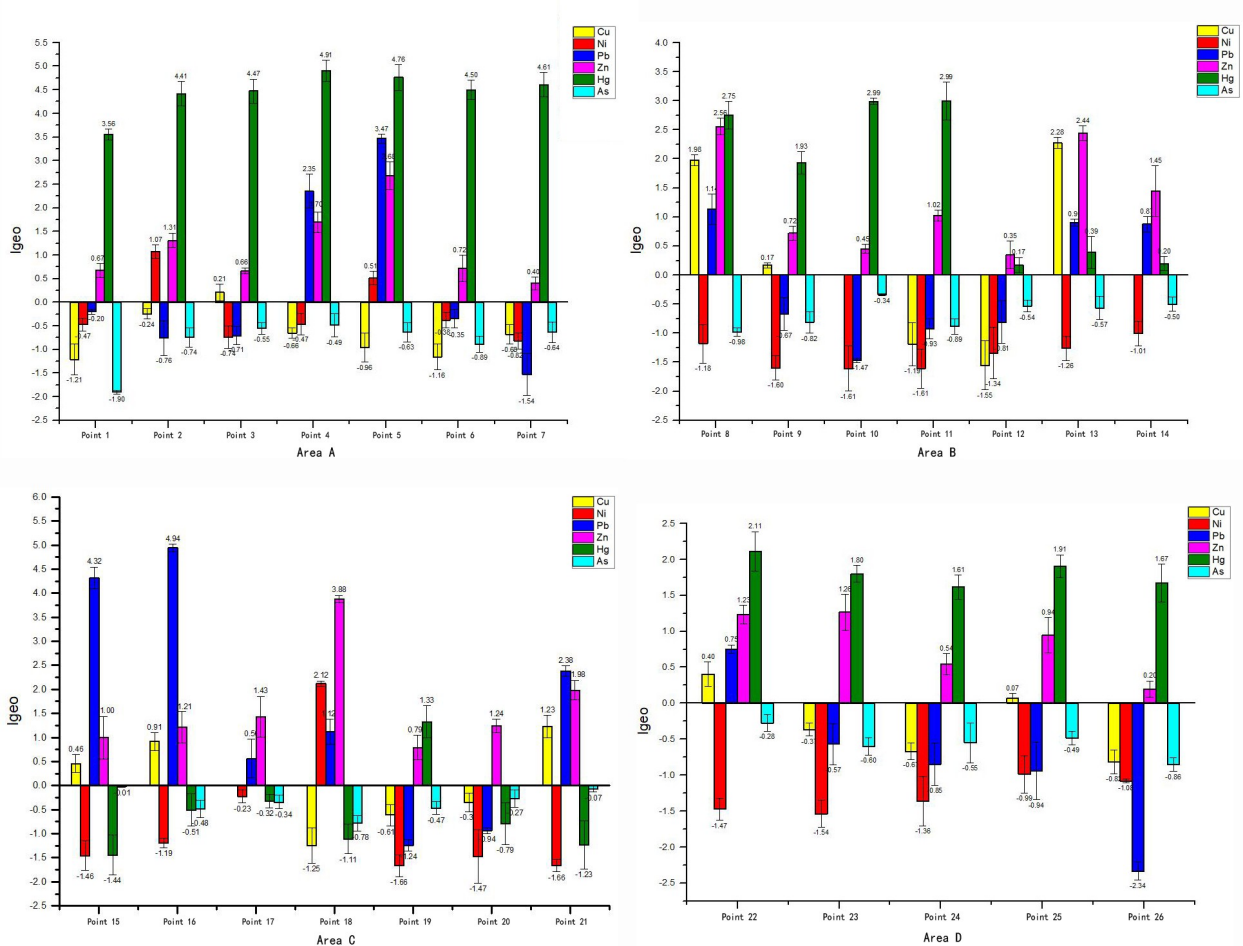
I_geo_ distribution of heavy metals of each sample point in area A, B, C, D.

The results of I_geo_ are shown in Fig 3. The pollution of Cu, Ni, Pb, Zn and Hg existed and differed in degrees, among which Hg, Pb and Zn were comparatively serious, and the pollution happened in areas A, B, C and D at the same time. The average I_geo_ values of all points were arranged in the order of Hg (1.83) > Zn (1.26) > Pb (0.33), showing certain spatial differences. The I_geo_(Hg) of all seven sample points in area A were greater than 3.50, which belonged to strong pollution or extremely strong pollution, and the maximum value was 4.91. The I_geo_(Pb) of sample points 15 and 16 in area C were 4.32 and 4.94 respectively, which belonged to strong pollution extremely strong pollution, and they were significantly higher than other points. The maximum value of I_geo_(Zn) was 3.88 at point 18.

**Fig 4:**
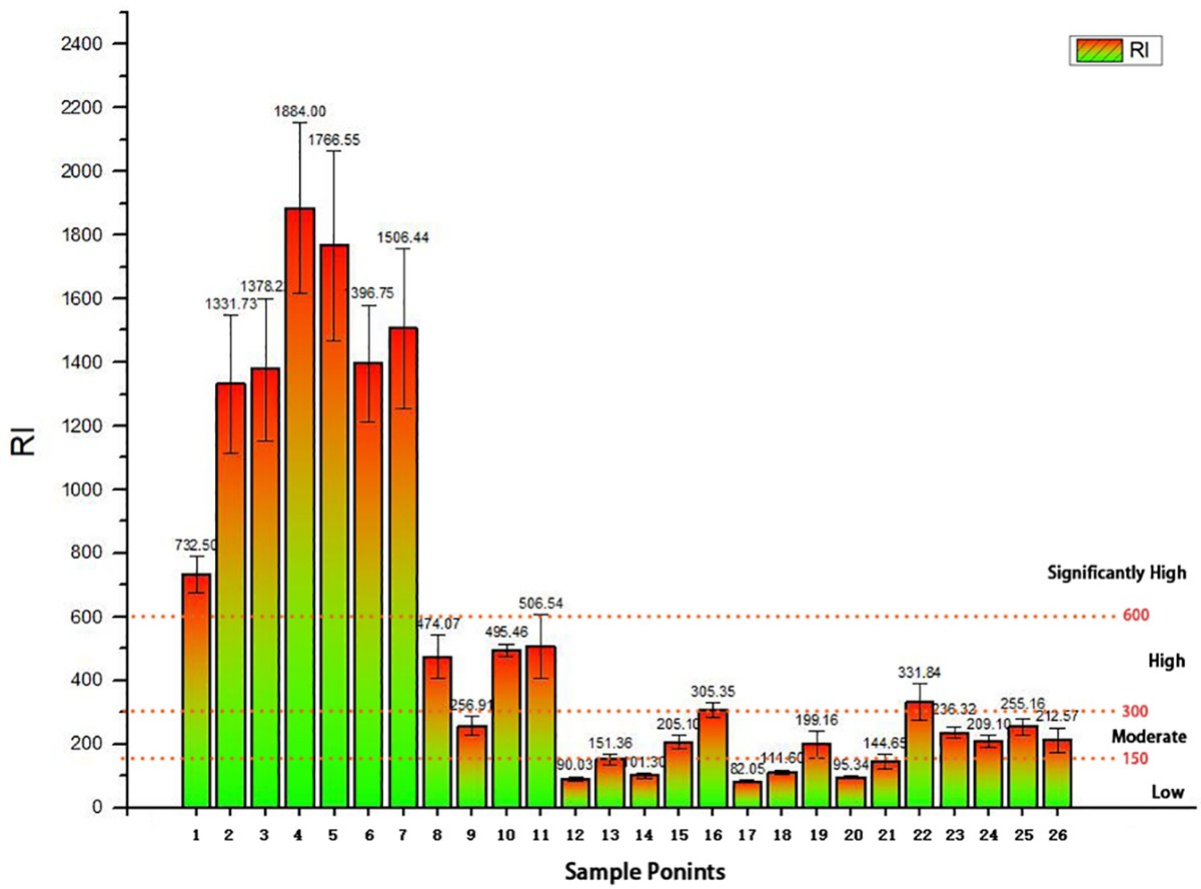
RI and comprehensive PER of each sample point.

The RI results are shown in Fig 4. The RI values of sample points (1–7) in area A were all greater than 600. The maximum of RI can reach 1884 in point 4, and the average value of RI in area A was 1428. It dicateed that their PERs were significantly high and obviously larger than other areas. Through the analysis of E_r_^i^, Hg is the main cause resulting in the significantly high level of PER in area A. The RI values of sample points 8, 10, 11 in area B were 474, 495 and 506 respectively. They were high risk points. Similarly, it mainly resulted from the ecological risk of Hg. The RI valus of sample point 16 in area C was slightly higher than 300, which due to the high concentration of Pb. In area D, the high level of PER at point 22 was mainly affected by Hg. Under the consideration of negative impact of different heavy metals on environment, areas A, B, C and D had been polluted in varying degrees. The average PER of different areas were in the order of A (1428) > B (297) > D (249) > C (163).

## 4. Discussion

### 4.1. Pollution sources

According to the assessment resutls, the contamination of Hg, Pb and Zn was relatively severe, especially Hg. Generally the heavy metal pollution of Hg existed in the whole research areas with its average value of I_geo_(Hg) was 1.83. Chlor alkali industry is one of the main anthropogenic sources of Hg pollution in urban areas [30,31]. Acetylene method has been widely used in the raw material production of chlor alkali industry. The catalyst containing Hg is indispensable in the chemical process. As a result, the waste catalyst, activated carbon, acid and water will cause potential Hg pollution to the environment. Through portable quick detection and artificial identification, old buildings (abondoned), chemical facilities (abondoned) and open spaces were selected as different research areas. Some of these areas were listed as potential pollution areas by the government, which were consistent with the research results. Sample points 1–11 were close to chemical reaction facilities, which showed significantly high concentration of Hg. Area D is now open space, which used to be a big chemical plant. Although the buildings and facilities had been demolished, there still existed potential heavy metal pollution of Hg. Hg and its compounds have strong biological toxicity and bioaccumulation, which has potential carcinogenic risk.

**Fig 5:**
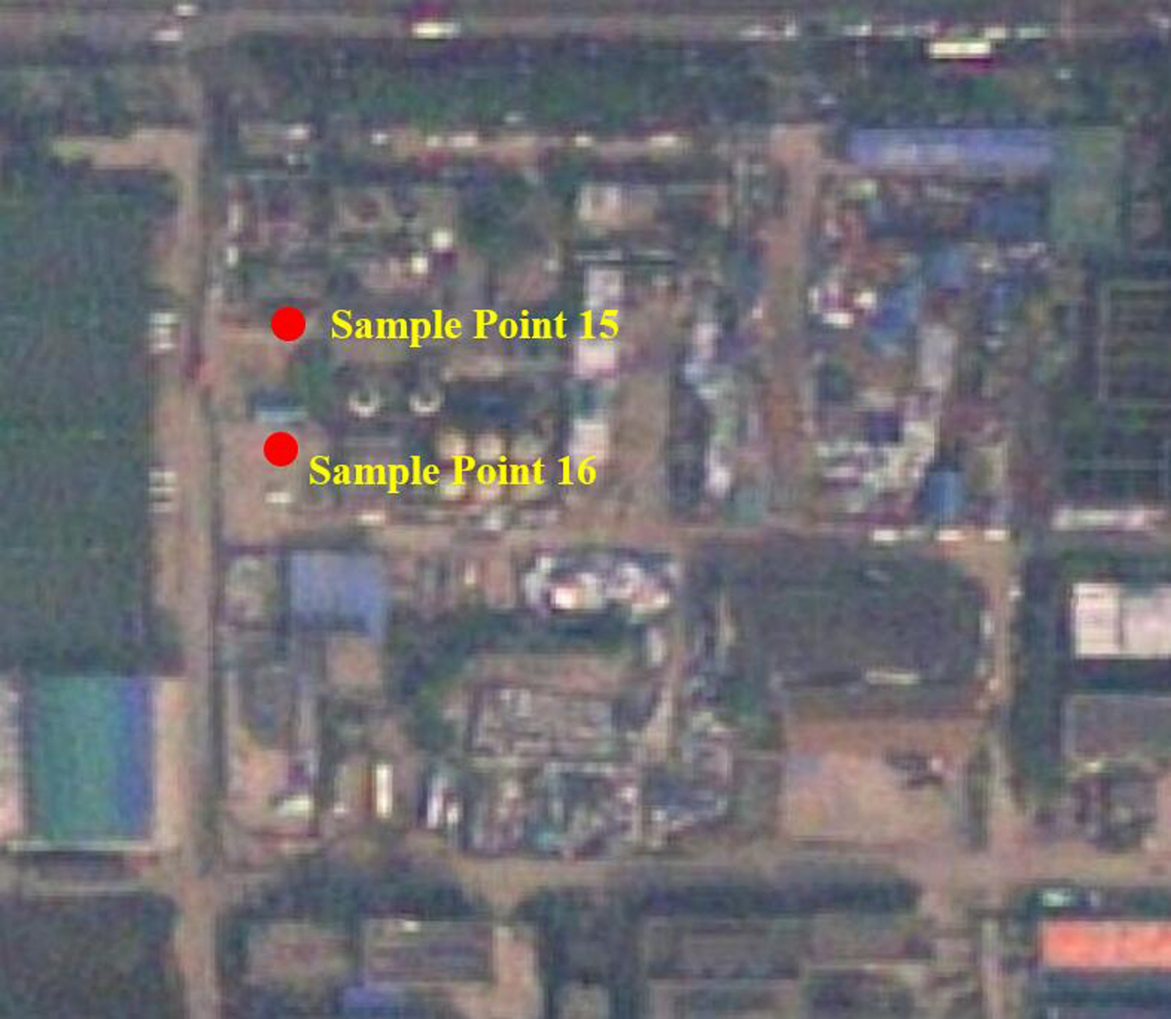
Satellite image of area C (The map was generated using free and open access data sources from National Platform for Common Geospatial Information Services. http://www.tianditu.gov.cn/).

Heavy metal pollution of Pb in urban soil mainly comes from the products of fossil fuel combustion. Particularly, transportation and automobile exhaust are the main causes of soil Pb pollution in the process of urbanization [16,32]. In general, the concentration of Pb is directly proportional to the traffic flow [33]. The abandoned site in this research was still being used for other purposes including public parking, vehicle repair, logistic warehousing and so on, which leads to the pollution of Pb. From Fig 5, it can be found that sample point 15 and 16 were now being used as intensive parking area. The average of I_geo_(Pb) was 0.33, which was not obvious. However, the values of I_geo_(Pb) in sample point 15 and 16 were all larger than 4.00. The pollution of Pb at these points was apparently more serious than others. The I_geo_(Pb) in sample points 4, 5, 8, 13–18, 21, 22 were all greater than 0.00. Through remote sensing and field investigation, it could be found that automobile activities at these points, including public parking and vehicle repair, were more active and frequent. By comparing the intensity and frequency of automobile activities with the concentration of Pb, the positive correlation between automobile activities and the concentration of Pb in soil has been confirmed. Additionally, the pollution distribution of Zn was similar to that of Pb. In this research, the I_geo_(Zn) of a sample was greater than 1.00 when the I_geo_(Pb) was greater than 0.00. Automobile exhaust and tire abrasion will lead to the accumulation of Pb and Zn in soil. Therefore, the concentration of Pb and Zn in soil was directly affected by human activities in scope and form. Besides, the pollution of Pb happened along with the chemical production, such as at sample point 4 and 21. They were close to chemical reaction facilities and suffering from the pollution of Pb according to the result of I_geo_. To sum up, the pollution sources of research areas were various including industrial legacy and unsustainable land reuse. We should not only control the original chemical pollution, but also prevent the secondary pollution in the process of land redevelopment.

### 4.2. Brownfield development based on man-land interrelations

**Fig 6:**
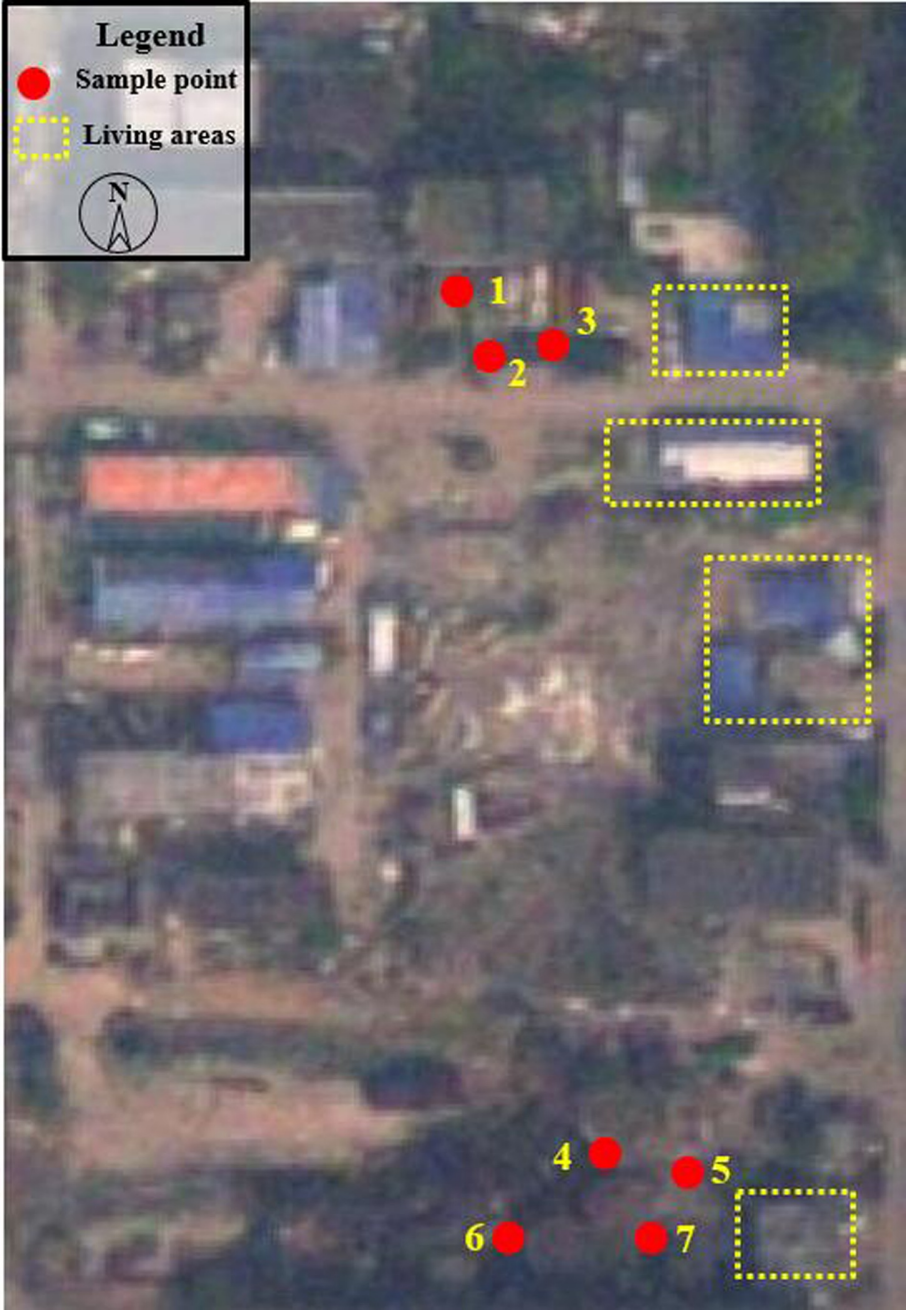
Satellite image of area A (The map was generated using free and open access data sources from National Platform for Common Geospatial Information Services. http://www.tianditu.gov.cn/).

Urban development is a dynamic process. In the process of rapid urban expansion, land use patterns are also changing [34,35]. Although the ratio of urbanization has been improved, the urban ecological environment is deteriorating and the urban function is deficient. Within this context, the development of urban brownfield is inefficient and unsafe. In this study, the research area not only undertaked part of the urban functions, such as public parking, logistics and warehousing, but also became the living place for some low-income people. According to the analysis above, all sample points in area A had great risk for ecological environment with their RI were significantly high. However, it can be found that there were still places where people were active through on-site survey. It seemed like a small community involving kinds of activities, such as living and catering (Fig 6). It was a spontaneous behaviour. There were great threats to the ecological environment and human health. According to Table 8, in recent years, the surrounding land of the research areas had gradually become residential areas. The relationship between human and land was becoming more complicated. People became more sensitive to the way land was used. The improvement of ecological environment quality is the core of urban development. In the development process of brownfield, we should pay attention to the connection with the surrounding land. The development of brownfield is important to the improvement of urban functions and land economic benefits. More importantly, the development of brownfield is of great significance to ensure land security and regional ecological environment construction. On the one hand, we should fully protect and remediate the land pollution, and it is necessary to avoid the damage of ecological environment to the surrounding areas. On the other hand, the development of brownfield needs to start from people's demand, especially the surrounding residents. From the view of urban administrator, public participation of stakeholder will be helpful to make more reasonable site land use planning scheme instead of a spontaneous one.

**Table 8:** Land use changes from 2002 to 2018 around the site.

Time of Satellite image	Spatial relationship with the research areas			
	East	South	West	North
**2002**	industrial plants and affiliated community	rural areas	rural areas	industrial plants
**2009**	steel warehouse	rural areas and a part of steel warehouse	rural areas	demolishing industrial plants
**2013**	demolishing steel warehouse	demolishing steel warehouse and the rural areas became residential areas	sewage-treatment plant expansion	residential areas
**2018**	residential areas	residential areas	residential areas	residential areas

*refer to the Official Investigation Report of Xi'an Chlor Alkali Chemical Plant, 2019, redrawn by author.

For cities, land resource is valuable and scarce. Brownfield is out of control for a long time owing to the difficulty of pollution identification, control and development, which is a waste of urban land resources. According to the past and present land use pattern, well-directed identification of pollution needs to be implemented. The brownfield should be classified based on different levels of harm to the ecological environment. Use of site-specific recommendations to suit different land use pattern to local conditions will promote human-land harmonious development. In the area with high potential risk, activities that closely related to human health should be avoided, such as living and catering. In the area without pollution or relatively slight pollution, these places can take on part of urban functions, such as urban green land, public parking and open space. It must be noted that the spatial scale and pattern need be controlled strictly in order to avoid secondary pollution

## 5. Conclusion

Through on-site portable quick detection and laboratory chemical analysis, totally 26 sample points in four different areas (A, B, C, D) were analyzed. Hg, Zn, and Pb were the main pollutants with the mean values of 0.81 mg·kg^-1^, 140.88 mg·kg^-1^ and 205.98 mg·kg^-1^, respectively. Different assessment methods including PI, I_geo_, PER indicated the pollution condition between each sample point was quite different in research areas. The PER of area A reached significantly high level while the PERs of other areas were moderate level. This was mainly due to the high concentration of Hg in area A. The I_geo_ results indicated the pollution of Hg was extensive in research areas. 76.9% of the sample points were contaminated by Hg. The I_geo_(Hg) of sample points 1–7 in area A were all greater than 3.50. The high concentration of Hg was mainly from historical pollution produced by chemical plant. The mean value of I_geo_(Pb) was only 0.33, however, the pollution of Pb was significant in the sample points where automobile activities are frequent and active. Additionally, the pollution distribution of Zn was similar to that of Pb. This rule was closely related to automobile exhaust and tire abrasion.

In China, people lack of systematic understanding of brownfield. In general, the development of brownfield is inefficient and unsafe, which is of great risk to urban ecological environment. When people utilize the land in different ways, it also gives people different ecological feedbacks. And then, the changes of ecological environment will affect human activities conversely. This is an internal man-land relationship. They influence each other and develop in coordination. On the basis of fully understanding the land conditions, it is necessary to identify the land pollution types and pollution sources. As a result, ecological hazards can be minimized and scientific development mode can be formed.

## Supporting information

S1 Data Complete primary data file. Please see text for explanation of the principal data collected from the samples.
(XLSX)
